# Helminths and Cancers From the Evolutionary Perspective

**DOI:** 10.3389/fmed.2018.00090

**Published:** 2018-04-16

**Authors:** Larissa L. S. Scholte, Marcelo A. Pascoal-Xavier, Laila A. Nahum

**Affiliations:** ^1^Instituto René Rachou, Fundação Oswaldo Cruz (FIOCRUZ), Belo Horizonte, Brazil; ^2^Vice-Presidência de Pesquisa e Coleções Biológicas, Fundação Oswaldo Cruz (FIOCRUZ), Rio de Janeiro, Brazil; ^3^Departamento de Anatomia Patológica, Faculdade de Medicina, Universidade Federal de Minas Gerais, Belo Horizonte, Brazil; ^4^Faculdade Promove de Tecnologia, Belo Horizonte, Brazil

**Keywords:** parasite, microbiome, cancer, biomarkers, phylogeny, molecular evolution, evolutionary medicine, bioinformatics

## Abstract

Helminths include free-living and parasitic Platyhelminthes and Nematoda which infect millions of people worldwide. Some Platyhelminthes species of blood flukes (*Schistosoma haematobium, Schistosoma japonicum*, and *Schistosoma mansoni*) and liver flukes (*Clonorchis sinensis* and *Opisthorchis viverrini*) are known to be involved in human cancers. Other helminths are likely to be carcinogenic. Our main goals are to summarize the current knowledge of human cancers caused by Platyhelminthes, point out some helminth and human biomarkers identified so far, and highlight the potential contributions of phylogenetics and molecular evolution to cancer research. Human cancers caused by helminth infection include cholangiocarcinoma, colorectal hepatocellular carcinoma, squamous cell carcinoma, and urinary bladder cancer. Chronic inflammation is proposed as a common pathway for cancer initiation and development. Furthermore, different bacteria present in gastric, colorectal, and urogenital microbiomes might be responsible for enlarging inflammatory and fibrotic responses in cancers. Studies have suggested that different biomarkers are involved in helminth infection and human cancer development; although, the detailed mechanisms remain under debate. Different helminth proteins have been studied by different approaches. However, their evolutionary relationships remain unsolved. Here, we illustrate the strengths of homology identification and function prediction of uncharacterized proteins from genome sequencing projects based on an evolutionary framework. Together, these approaches may help identifying new biomarkers for disease diagnostics and intervention measures. This work has potential applications in the field of phylomedicine (evolutionary medicine) and may contribute to parasite and cancer research.

## Introduction

Helminths are polyphyletic, i.e., they have multiple origins over evolutionary time [e.g., see Ref. (1–4)]. They include free-living and parasitic species of Platyhelminthes (flatworms) and Nematoda (roundworms). Together, helminth parasitic species infect millions of people worldwide.

Infections by helminths may cause different types of cancers as shown by independent studies [reviewed in Ref. (5–9)]. This Mini Review is focused on Platyhelminthes. However, it is worth mentioning that some Nematoda are also related to human cancers [e.g., see Ref. (10, 11)].

The main goals of this Mini Review are to (1) summarize the current knowledge of human cancers caused by Platyhelminthes, (2) point out some helminth and human biomarkers identified so far, and (3) highlight the potential contributions of phylogenetics and molecular evolution to cancer research.

## Helminths and Human Cancers

Platyhelminthes include parasitic flatworms that infect millions of people worldwide. To date, some species are known to be involved in human cancers. Liver flukes causing cholangiocarcinoma include *Clonorchis sinensis* and *Opisthorchis viverrini*. Blood flukes causing different types of cancers include *Schistosoma haematobium* (urinary bladder cancer), *Schistosoma japonicum* (colorectal hepatocellular carcinoma), and *Schistosoma mansoni* (colorectal hepatocellular carcinoma). The first three species mentioned above are classified as Group 1 carcinogens by the International Agency for Research on Cancer (IARC) (12).

Excellent reviews have focused on the association between Platyhelminthes and cancers [e.g., see Ref. (6, 13)]. These studies emphasize chronic inflammation as a common pathway for the initiation and development of cancer. Besides, they detail the carcinogenic process for cholangiocarcinoma and squamous cell carcinoma highlighting the oncogenes activation, suppressor genes inactivation, and somatic mutations as key factors in the initiation and promotion of malignancy.

Complementarily and challenged by the questioning of Brindley and colleagues (5, 9), other studies proposed a new hypothesis: reactive metabolites of oxysterol-like and estrogen-like precursors of helminth origin represent genotoxins that mutate genes of epithelial cells lining the biliary tract and urinary bladder and initiate biliary duct cancer and squamous cell carcinoma of the bladder during opisthorchiasis and urogenital schistosomiasis. In addition, Brindley and Loukas (14) extended the hypothesis for helminth-specific metabolites and included growth factors that induce repair and angiogenesis (14).

However, given the insufficiency of these hypotheses to explain the occurrence of cancer in the smallest part of the parasitized patients, more recent studies explore the auxiliary role of gastric, colorectal, and urogenital microbiomes (15–19).

Thus, Plieskatt and colleagues (18) demonstrated that infection with *O. viverrini* led to changes in the microbial communities of the gastrointestinal tract, including the emergence of microbes in the biliary system, enlarging inflammatory and fibrotic responses originated during opisthorchiasis. Subsequently, Sripa and colleagues (19) hypothesized that co-infection with *Helicobacter* species induces epithelial and adenomatous hyperplasias in the biliary tract (19).

In parallel, Itthitaetrakool and colleagues (17) demonstrated that chronic infection by *O. viverrini* enhances bacterial diversity in the liver and promotes *Helicobacter pylori* growth (17). Other bacteria (Dietziaceae, Oxalobacteraceae, and Pseudomonadaceae) predominate in the cancer microbiome and enteric bacteria (Bifidobacteriaceae, Enterobacteriaceae, and Enterococcaceae) prevail in the *O. viverrini* microbiome, establishing a linkage with carcinogenesis (16).

In addition, Adebayo and colleagues (15) studied the urinary microbiome during *S. haematobium* infection and demonstrated that specific microorganisms are associated with both inflammation and host protection (15). They noted that Proteobacteria and Firmicutes dominated the microbiome of both non-infected persons and persons with urogenital schistosomiasis.

Together, these pieces of evidence strengthen the importance of a phylogenetic approach of helminth, microbiome, and cancer associations as factors in identifying biomarkers and developing diagnostic tools for cholangiocarcinoma, urinary bladder cancer, and other cancers.

## Biomarkers in Human Cancers

During the past years, the scientific community has contributed to the tremendous progress in cancer research, including the identification of biomarkers involved in human cancers caused by helminths (9, 20–23).

In this context, a better understanding regarding cancer pathogenetic evolution can provide a positive impact in clinical procedures, more specifically related to diagnosis and therapeutics. Alterations within cancer cells at the molecular level (DNA, mRNA, miRNA, proteins, lipids, and carbohydrates) can be used as “sentinels” for risk assessment, differential diagnosis, prediction of treatment response, prognosis determination, and also for monitoring disease progression.

As previously mentioned, the carcinogenic potential of some parasitic species of Platyhelminthes was previously described in opisthorchiasis, clonorchiasis, and schistosomiasis. Besides causing public health issues through parasitism with consequences to human populations, *C. sinensis* and *O. viverrini* can also lead to cholangiocarcinoma development (bile duct cancer), while *S. haematobium* has been related to squamous cell carcinoma of the urinary bladder [reviewed in Ref. (9)]. Although the mechanisms by which helminth infection initiate genetic lesions that may result in cancer are likely to be multifactorial and not completely understood, some potential biomarkers have been described (10, 22, 24–27).

Gouveia and colleagues (25) used liquid chromatography-mass spectrometry to analyze urine from patients with urogenital schistosomiasis, revealing catechol estrogen quinones (CEQ), CEQ-DNA-adducts, 8-oxo-7, and 8-dihydro-2′-deoxyguanosine (8-oxodG) metabolites, which were not described in the metabolome database of healthy human urine (25, 28). For instance, 8-oxodG is a known biomarker for DNA oxidative damage and its significantly higher expression in bladder cancer may characterize a clear evidence that urogenital schistosomiasis can lead to tumor development (22, 26). Recently, a proteomic analysis conducted by Bernardo and colleagues (29), studying urine samples from urogenital schistosomiasis-induced carcinogenesis, supported the hypothesis that most cancers are likely to originate with a *stimulus* (biological or chemical), followed by chronic inflammation, fibrosis, and changes in the cellular microenvironment that result in transition from normal to cancer cells (29).

In addition, increased levels of urinary b-glucuronidase, cyclooxygenase-2, and nitrosamines have been pointed as carcinogenic compounds that lead to DNA damage and, consequently, to events such as DNA strand breaks, mutations, and sister chromatid exchanges (24, 27). Furthermore, changes in oncogenes, such as p53, retinoblastoma protein, epidermal growth factor receptor, erb-b2 receptor tyrosine kinase 2 (ERBB2), and Kirsten rat sarcoma viral oncogene homolog (KRAS), have been observed and a combination of distinct markers with synergistic effects can be used to stratifying patients into different risk groups [e.g., see Ref. (30–32)].

Similar findings were obtained for *C. sinensis* and *O. viverrini* where parasite-derived molecules from long-lasting infections can lead to uncontrolled growth of host cells and result in cholangiocarcinoma, the most common biliary tract malignancy, with dismal prognosis [e.g., see Ref. (33–35)]. Distinct biomarkers have been shown to be associated with cholangiocarcinoma, such as the carbohydrate antigen CA-19-9, which is widely used for diagnosis, but lacks specificity (36), interleukin-6 involved in pathogenesis of advanced periductal fibrosis (37), reactive oxygen species (ROS), and reactive nitrogen species (38). Furthermore, Maeng and collaborators (39) pointed out that the expression of various lipid peroxidation products were elevated during the infection. For instance, 8-oxodG, which is product of DNA lesion, was initially detected in the nucleus of the inflammatory cells and in the biliary epithelial cells, creating an environment that favors the development of diseases such as cholangiocarcinoma. Moreover, cofactors including preferences for nitrosamines-rich foods can exacerbate risk factors for cholangiocarcinoma [e.g., see Ref. (40)].

Messina and colleagues (10) have investigated the potential association between the *Anisakis pegreffii* (Nematoda: Anisakidae) and malignancy by evaluating molecular biomarkers related to stress response, oxidative stress, inflammation, and apoptosis (10). Their findings show that *in vitro* cell response to *Anisakis* products results in increased production of ROS, activation of kinases, strong upregulation of Hsp70, and elevated induction of p53, which lead to inflammation and DNA damage.

It is clear that distinct helminths may act as carcinogens leading to cancer development in humans. By contrast, closely related parasites are classified as possibly carcinogenic to humans (Group 2B, e.g., *S. japonicum*) or not classifiable as carcinogenic to humans (Group 3, e.g., *S. mansoni*) suggesting that certain tissues/organs are more prone to infection-induced malignancy (14).

Altogether, studies involving parasite-specific metabolites and proteins can open new frontiers for the development of helminth-induced cancer biomarkers for diagnosis, prognosis, and treatment. Although it is clear the potential value that the evolutionary perspective can add to molecular approaches in cancer treatment, more research is needed to increase our knowledge regarding the relationships among biomarkers, oncogenesis, and stages of different cancers.

## Phylogenetics and Evolution Studies

Phylogenetics and molecular evolution studies have been applied to a broad range of technological and scientific areas (41–46). However, these approaches have been overlooked in human cancers caused by helminths.

The question of whether evolutionary processes in cancers are driven by natural selection and adaptation or by other processes, such as founder effect and genetic drift, remain under debate (41, 46, 47). Fortunato and colleagues (47) propose convergent evolution through natural selection as the only satisfying explanation both for how diverse cancers have enough in common and why this convergent condition becomes life-threatening (47).

Several applications of the evolutionary framework including different methods and tools may contribute to cancer research. Main steps in molecular phylogenetics include homology search, sequence alignments, tree reconstruction, and tree annotation [cf. (45, 48)]. Searching for potential homologs through sequence similarity searches is a common practice. However, several errors might arise due the limitations or misinterpretation of the results obtained by this procedure [e.g., see Ref. (49, 50)]. Ideally, different approaches should be combined to have more accurate and robust results.

Different methods and several tools for sequence alignment and tree reconstruction are available [cf. (48)]. Tree annotation might include taxonomic information, geographic localization, epidemiological information, experimental characterization, etc. [cf. (45)].

As mentioned in the Section “Introduction,” helminths are known to be polyphyletic [e.g., see Ref. (1–4)]. The most well-supported hypothesis suggests that both Nematoda and Platyhelminthes are believed to have multiple origins over evolutionary time. Moreover, some helminths are considered to correspond to different strains or cryptic species such as *O. viverini* [cf. (51)]. Furthermore, helminths have complex life cycles including multiple hosts and vectors.

Together, these observations make the study of helminths very challenging. In order to better understand the interactions among helminths and their hosts and vectors and ultimately make predictions to improve cancer research, it is necessary to focus on studies including phylogenetics, phylogeography, and phylodynamics, among other approaches (41, 47, 51).

Evolutionary biology combined to evolutionary epidemiology may significantly overcome the challenges aforementioned and contribute to a better understanding of processes (co-evolution, natural selection, genetic drift, mutations, adaptations, etc.) that shape the evolution of helminths, humans, and cancers (41, 51).

In this context, research and control of liver flukes in Southeast Asia from the evolutionary perspective has been reviewed (51). This work illustrates how the evolutionary framework may improve our understanding of helminth transmission dynamics, helminth–host relationships (co-evolution, etc.), cancer etiology, etc. These authors also organized an evolutionary biology research agenda to address different questions regarding the biology of host–parasite interactions, parasite adaptations, chemotherapy, cancer prevention, etc (51). The potential impacts of such research include improvements in clinical studies and evolutionary epidemiology, among others.

Microbiomes have been investigated through metagenomic studies revealing that parasite infections lead to inflammation and disease development. The helminth microbiota interacts with the host potentially inducing such processes (15–19). A research agenda focused on the evolutionary relationships of the microbiota of different helminths and humans will shed light on cancer biology and guide new intervention strategies.

To our knowledge, biomarkers discussed in this article have not been analyzed from an evolutionary perspective. Therefore, it is critically important to apply the evolutionary thinking to evaluate their relationships with other genes, gene products, and molecules in the cellular and extracellular environments that might be involved in cancer development and progression. Biomarkers should be experimentally characterized in detail in order to support a rational design of potential targets for cancer diagnostics and treatment.

Sequencing data generated by the studies aforementioned include a majority of genes and proteins whose functions are not experimentally characterized. These genes and proteins are annotated as predicted, hypothetical, unknown, etc. The evolutionary framework has a tremendous power in analyzing sequence data and making function predictions [e.g., see Ref. (52–56)].

Studies applying phylogenetics to improve functional prediction and characterization of genes and proteins that play key roles among human parasites have been described [e.g., see Ref. (52–56)]. These studies illustrate the strengths of homology identification and function prediction of uncharacterized proteins from parasite genome sequencing projects by using phylogenetics. Similar approaches could be applied to study helminth and human biomarkers involved in cancers.

Together, phylogenetics and molecular evolution studies may contribute to identify helminth and human biomarkers in the different types of cancers aforementioned. Besides providing a robust framework for functional prediction and rational design of their experimental characterization, these approaches may contribute to improve personalized treatment to avoid cancer progression and drug resistance [cf. (46)].

## Conclusion

Helminth infections cause different types of human cancers such as cholangiocarcinoma, colorectal hepatocellular carcinoma, squamous cell carcinoma, and urinary bladder cancer. Different biomarkers have been suggested as involved in cancers caused by helminth infection. However, the detailed mechanisms of human cancer development remain unresolved. Phylogenetics and molecular evolution studies of parasite and human genes and proteins may help identifying new biomarkers for disease diagnostics and intervention measures. Together, this work has applications in the field of phylomedicine, i.e., evolutionary medicine, and may contribute to the understanding parasite and cancer research (41, 42, 47, 51).

## Author Contributions

All authors have contributed equally to writing the manuscript. The final version was approved by all authors.

## Conflict of Interest Statement

The authors declare that the research was conducted in the absence of any commercial or financial relationships that could be construed as a potential conflict of interest.
